# Postprandial triglyceride-rich lipoproteins as predictors of carotid atherosclerosis in individuals with normal fasting lipid profiles: a prospective follow-up study

**DOI:** 10.3389/fendo.2025.1502792

**Published:** 2025-02-25

**Authors:** Yilin Hou, Peipei Tian, Guangyao Song, An Song, Dandan Liu, Zhimin Wang, Yihe Shi, Yale Tang, Xiaoyu Wang, Luxuan Li, Luping Ren

**Affiliations:** ^1^ Department of Internal Medicine, Hebei Medical University, Shijiazhuang, Hebei, China; ^2^ Department of Endocrinology, Hebei General Hospital, Shijiazhuang, Hebei, China; ^3^ Hebei Key Laboratory of Metabolic Diseases, Hebei General Hospital, Shijiazhuang, Hebei, China; ^4^ Department of Endocrinology, Key Laboratory of Endocrinology, National Commission of Health, Peking Union Medical College Hospital, Chinese Academy of Medical Science, Beijing, China

**Keywords:** atherosclerotic cardiovascular disease, carotid atherosclerosis, remnant cholesterol, triglycerides, postprandial, oral fat tolerance test

## Abstract

**Background:**

Carotid atherosclerosis (CAS), a key precipitator of cardiovascular incidents, is linked to postprandial triglyceride-rich lipoproteins (TRL), as reflected by elevated triglycerides (TG) and remnant cholesterol (RC). This study explores the oral fat tolerance test (OFTT) for its predictive value in CAS, using postprandial TRL levels as a diagnostic biomarker.

**Methods:**

A total of 107 volunteers with normal fasting lipid profiles and no CAS at baseline were included. They received an OFTT after consuming a meal containing 60% fat (1500 kcal). Serum lipid profiles were monitored at fasting and 2, 4, 6, 8, and 10 h postprandially. The participants were categorized into postprandial normotriglyceridemia and postprandial hypertriglyceridemia groups based on their postprandial TG levels. After a 6-year follow-up, fasting lipid profiles and CAS status were reassessed. The baseline fasting and postprandial lipid levels in the CAS and non-CAS groups were compared. Repeated-measures analysis of variance was used to analyze the postprandial lipid profiles across different groups. Logistic regression models were constructed to assess the effects of postprandial TG and RC levels on CAS incidence.

**Results:**

The incidence of CAS in the postprandial hypertriglyceridemia group was 66.0%, which was significantly higher than the 13.3% observed in the postprandial normotriglyceridemia group (*P* < 0.001). In the CAS group, postprandial TG and RC levels peaked 4 h after a high-fat meal and did not return to fasting levels, even after 10 h. The levels of 4h-postprandial TG (TG_4h_), maximum postprandial TG (TG_max_), 4h-postprandial RC (RC_4h_), and maximum postprandial RC (RC_max_) were significantly higher in the CAS group than in the non-CAS group (*P* < 0.05). At baseline, TG_4h_ (*P* < 0.001), TG_max_ (*P* = 0.006), RC_4h_ (*P* < 0.001), and RC_max_ (*P* = 0.003) were statistically significant predictors of CAS, whereas fasting TG (*P* = 0.200) and fasting RC (*P* = 0.200) were not significantly associated with CAS.

**Conclusion:**

The standardized OFTT has predictive value for CAS, and elevated TRL levels after a high-fat meal in individuals with normal fasting lipid profiles may serve as an early marker for CAS.

## 1 Introduction

Atherosclerotic cardiovascular disease (ASCVD) poses a major global threat to human health. Carotid atherosclerosis (CAS), an early indicator of ASCVD, has attracted considerable attention due to its significance as a marker of systemic atherosclerosis. Identifying and managing CAS progression is essential for reducing ASCVD risk (1). In 2020, an estimated 28% of individuals worldwide aged 30–79 years had CAS, with the highest prevalence observed in China and other Western Pacific countries (2). A Chinese survey of individuals over 20 years of age reported a CAS prevalence as high as 26.2% (3). Early signs of CAS typically include increased carotid intima-media thickness (cIMT), which may progress to carotid plaque formation, arterial stenosis, or occlusion. Carotid ultrasonography, a noninvasive screening tool, is widely employed to assess CAS and identify high-risk populations effectively (4).

Dyslipidemia is a risk factor for ASCVD, and lipid management plays a critical role in its prevention and treatment. Triglycerides (TG) are primarily transported in the body by TG-rich lipoproteins (TRL), such as chylomicrons, very-low-density lipoproteins, and their metabolic remnants, collectively known as remnant cholesterol (RC). RC, characterized by high cholesterol content and small particle size, can penetrate the vascular endothelial barrier, infiltrate the arterial wall, and be engulfed by macrophages. This process leads to foam cell formation and promotes atherosclerotic plaque development (5). RC’s atherogenic potential may surpass that of low-density lipoprotein cholesterol (LDL-C), making it a valuable supplementary marker for assessing cardiovascular disease risk. Consequently, TRL have become a major focus of global research. Reflecting advances in TG metabolism, the 2021 European Atherosclerosis Society consensus redefined fasting TG levels, classifying levels below 1.2 mmol/L as optimal, 1.2–1.7 mmol/L as borderline, and levels of 1.7 mmol/L or higher as elevated (6).

Postprandial lipid fluctuations significantly influence ASCVD development. Epidemiological studies suggest that non-fasting TG levels measured within 8 h after a meal are better predictors of ASCVD risk than fasting TG levels (7). However, capturing accurate TG peaks in non-standardized dietary conditions is challenging. The oral fat tolerance test (OFTT) addresses this limitation by dynamically monitoring postprandial lipid levels following the consumption of a standardized high-fat meal. The OFTT effectively identifies individuals with postprandial hypertriglyceridemia (PH) even when normal fasting lipids levels are normal. It also captures TG peaks while controlling factors such as dietary habits, fasting duration, and physical activity (8, 9).

Our previous research has shown that postprandial dyslipidemia induced by a single high-fat meal is associated with several metabolic dysfunctions, including fatty liver disease, inflammatory responses, insulin resistance, and abnormal apolipoprotein secretion (10–18). Apolipoprotein B (ApoB), a structural protein for LDL and very low-density lipoproteins, is implicated in atherosclerosis progression (19). Similarly, apolipoprotein C3 (ApoC3) inhibits lipoprotein lipase activity, delaying TRL clearance and increasing plasma levels of TG and cholesterol. This mechanism promotes the accumulation of atherogenic lipoprotein remnants during atherosclerosis (6). Additionally, interleukin-6 (IL-6) and high-sensitivity C reactive protein (hs-CRP), both positively correlated with serum TG levels, play roles in the shared pathophysiological mechanisms of lipid metabolism disorder and atherosclerosis (20).

Despite these insights, long-term follow-up studies of the Chinese population after an OFTT are limited. This study aimed to identify individuals with normal fasting lipid levels and no evidence of CAS during routine physical examinations, differentiate postprandial lipid states using the OFTT, and conduct long-term follow-ups. Our objectives were to explore the association between postprandial lipid changes and CAS, evaluate the potential value of the OFTT in early CAS prevention, and provide valuable longitudinal research data.

## 2 Materials and methods

### 2.1 Study population

This follow-up study, conducted in 2024, included 107 adult participants who underwent an OFTT at Hebei General Hospital, China, in 2018. All participants provided informed consent, and the study was approved by the Hebei General Hospital Ethics Committee (2018-02). This trial was registered with the Chinese Clinical Trial Center (ChiCTR1800019514).

Inclusion criteria required participants to have baseline fasting TG < 1.7 mmol/L, total cholesterol (TC) < 5.2 mmol/L, and LDL-C < 3.4 mmol/L (21). Additionally, baseline physical examinations had to show a cIMT < 1.0 mm, no localized thickening, and no plaque formation (4).

Exclusion criteria included self-reported history of pregnancy, cardiovascular disease, diabetes, thyroid disease, liver or kidney disease, cancer, or other serious illnesses. Participants who smoked, quit smoking within the past 3 years, or consumed alcohol more than once per week in the previous year were excluded, as were those who had taken oral hypoglycemic agents, lipid-lowering drugs, antihypertensive medications, or other drugs affecting blood lipid levels within the past year. Exclusion also applied to individuals experiencing stress conditions, such as infection, surgery, or major trauma, within the previous month.

### 2.2 Collection of baseline and follow-up data

General information, including age and sex, was recorded. Trained personnel performed physical examinations, measuring height and weight in order to determine their body mass index (BMI). Measurements were taken of the waist and hip circumferences to calculate the waist-to-hip ratio. Blood pressure measurements included systolic blood pressure (SBP) and diastolic blood pressure (DBP).

A 7600 automatic biochemical analyzer (Hitachi Instruments Ltd., Japan) was used to measure serum TC, TG, high-density lipoprotein cholesterol (HDL-C), LDL-C, blood glucose, Apo B, and hs-CRP. LDL-C was calculated using the Friedewald equation [LDL-C = TC − (HDL-C) − TG/2.2] for TG levels ≤ 4 mmol/L; for TG levels > 4 mmol/L, measured LDL-C values were used (22). RC was calculated as RC = TC –(HDL-C) – (LDL-C), and non-HDL-C was calculated as non-HDL-C = TC – (HDL-C). Glycated hemoglobin (HbA1c) was measured using a VARIAN II hemoglobin analyzer (Bio-Rad Laboratories, USA). Apo C3 and IL-6 levels were determined using enzyme-linked immunosorbent assay kits from R&D Systems, USA, and Elabscience, China, respectively.

### 2.3 Definition of optimal fasting TG level

Participants were categorized based on an optimal fasting TG level of 1.2 mmol/L (6). Group A included those with fasting TG levels < 1.2 mmol/L; in contrast, Group B included participants with fasting TG levels between 1.2 and 1.7 mmol/L.

### 2.4 Definition of PH

Participants were instructed to avoid vigorous physical activity and high-fat or high-protein meals for 1 week before the trial. They also fasted for at least 8 h before the test. On the trial day, participants consumed a standardized test meal at the hospital between 7:00 and 8:00 AM. The test meal was prepared as specialized energy bars formulated by a professional nutritionist. Each bar consisted of 97 g of peanut oil (Luhua Group Co., Ltd., China), 86 g of flour (Wilmar International Limited, China), and 86 g of whey protein (Nestle Health Science Co., Ltd., United States). The meal’s nutritional content included 99.2 g of fat (17.5 g saturated fat, 43.1 g monounsaturated fat, and 37.5 g polyunsaturated fat), 66.9 g of carbohydrates, and 83.2 g of protein, amounting to approximately 1,500 kcal. Participants finished the meal within 10 min and refrained from consuming any food or beverages (except water) for 10 h postprandially. Vigorous physical activity was also prohibited during this period. Blood samples were collected every 2 h for 10 h postprandially, and the serum was stored at -80°C (Haier Group, China) for subsequent testing.

Using the 2010 European criteria (8), PH was defined as any postprandial TG level > 2.5 mmol/L. In contrast, postprandial normal (PN) was defined as postprandial TG levels consistently ≤ 2.5 mmol/L.

### 2.5 Assessment of atherosclerosis

Carotid ultrasound assessments were conducted by experienced physicians at Hebei General Hospital using the EPIQ 7C color ultrasound diagnostic system (Philips Ultrasound, Inc., FL, USA). cIMT was measured as the average thickness of the intima-media layer of the bilateral common carotid arteries, their bifurcations, and the internal carotid arteries at three sites. CAS was identified based on an average cIMT ≥ 1.0 mm and/or the presence of carotid atherosclerotic plaques. Plaques were defined as localized structures extending into the arterial lumen by ≥ 0.5 mm, vascular lumen thickness exceeding 50% of the surrounding cIMT, or a cIMT ≥ 1.5 mm (2, 4).

### 2.6 Statistical analysis

Statistical analyses and graphing were performed using R software (version 4.4.1) and GraphPad Prism 8. Descriptive statistics were used to analyze baseline and follow-up data, as well as changes in blood lipid levels during the OFTT, categorized by PH and CAS. Quantitative data with normal distributions are expressed as the mean ± standard deviation; in contrast, non-normally distributed data are expressed as the median (interquartile range). Categorical data are presented as counts and percentages (n, %). Independent sample *t*-tests were used to compare quantitative data between groups, and chi-squared tests were used for categorical data. Two-way repeated-measures analysis of variance was applied to assess the effects of time and group on blood lipid levels during the OFTT, with pairwise comparisons conducted using the Šidák method. Postprandial blood lipid levels were represented by the maximum values of TG and RC at any time postprandially (TG_max_ and RC_max_, respectively), as well as TG and RC at 4 h postprandially (TG_4h_ and RC_4h_). Univariate and multivariate logistic regression analyses were performed to evaluate associations between independent variables and CAS, with subgroup analyses based on age, sex, and BMI. Odds ratios (OR) and 95% confidence intervals (95% CI) were calculated. Statistical significance was defined as *P* < 0.05, with all tests conducted as two-tailed.

## 3 Results

### 3.1 Clinical characteristics of participants with different postprandial TG levels

This study included 107 participants who underwent follow-up after an OFTT, categorized into PN (n = 54) and PH (n = 53) groups. **Table 1** presents the baseline and follow-up clinical characteristics of participants with different postprandial TG levels. The cohort comprised 52 men and 55 women, with a baseline age of 47 (39–53) years and a follow-up age of 53 (45–59) years.

**Table 1:** Clinical characteristics of the participants with different postprandial TG levels.

Variables	Baseline				Follow-up			
	Total (n = 107)	PN (n = 54)	PH (n = 53)	*P* value	Total (n = 107)	PN (n = 54)	PH (n = 53)	*P* value
Sex (Men, %)	52 (48.6)	19 (35.2)	33 (62.3)	0.009	52 (48.6)	19 (35.2)	33 (62.3)	0.009
Age (year)	47 (39, 53)	47 (38, 51)	47 (39, 54)	0.321	53 (45, 59)	53 (44, 57)	53 (45, 60)	0.321
SBP (mmHg)	121.54 ± 14.28	120.28 ± 15.06	122.83 ± 13.47	0.358	126.34 ± 15.01	124.33 ± 15.65	128.38 ± 14.19	0.165
DBP (mmHg)	75.00 (70.00, 81.00)	75.00 (70.00, 80.00)	78.00 (70.00, 82.00)	0.183	78.00 (72.00, 84.00)	77.00 (72.25, 82.75)	78.00 (72.00, 89.00)	0.263
BMI (kg/m^2^)	25.40 (23.65, 27.40)	24.55 (21.55, 25.80)	26.60 (24.50, 29.20)	< 0.001	25.70 (24.11, 28.03)	25.19 (22.35, 26.81)	26.89 (25.18, 28.52)	0.001
Waist-to-hip ratio	0.87 ± 0.07	0.84 ± 0.07	0.89 ± 0.07	< 0.001	0.87 ± 0.07	0.84 ± 0.06	0.89 ± 0.06	< 0.001
FBG (mmol/L)	5.34 ± 0.49	5.25 ± 0.42	5.43 ± 0.55	0.059	5.34 (4.99, 5.83)	5.25 (4.99, 5.54)	5.50 (5.01, 6.13)	0.051
HbA1c (%)	5.50 (5.40, 5.70)	5.40 (5.32, 5.70)	5.50 (5.40, 5.75)	0.291	5.50 (5.40, 5.75)	5.50 (5.30, 5.68)	5.60 (5.40, 5.90)	0.005
TG (mmol/L)	1.05 ± 0.31	0.89 ± 0.28	1.22 ± 0.24	< 0.001	1.01 (0.78, 1.37)	0.81 (0.67, 1.01)	1.33 (1.10, 1.65)	< 0.001
RC (mmol/L)	0.48 ± 0.14	0.41 ± 0.13	0.55 ± 0.11	< 0.001	0.46 (0.35, 0.62)	0.37 (0.30, 0.46)	0.60 (0.50, 0.75)	< 0.001
TC (mmol/L)	4.17 (3.68, 4.60)	4.07 (3.46, 4.58)	4.24 (3.97, 4.61)	0.178	4.56 ± 0.80	4.40 ± 0.83	4.74 ± 0.75	0.028
LDL-C (mmol/L)	2.43 (2.03, 2.84)	2.34 (1.83, 2.83)	2.47 (2.32, 2.86)	0.097	2.71 ± 0.65	2.59 ± 0.64	2.84 ± 0.65	0.047
HDL-C (mmol/L)	1.18 (1.07, 1.37)	1.25 (1.15, 1.46)	1.12 (1.04, 1.21)	< 0.001	1.28 (1.10, 1.56)	1.35 (1.20, 1.64)	1.20 (1.02, 1.46)	0.008
Non-HDL-C (mmol/L)	2.97 (2.42, 3.38)	2.73 (2.23, 3.32)	3.04 (2.83, 3.45)	0.006	3.22 ± 0.70	2.98 ± 0.69	3.46 ± 0.63	< 0.001
cIMT (mm)	0.55 (0.45, 0.65)	0.52 (0.40, 0.60)	0.60 (0.50, 0.75)	0.005	0.75 (0.60, 0.90)	0.65 (0.50, 0.80)	0.80 (0.65, 1.10)	< 0.001

BMI, body mass index; cIMT, carotid intima-media thickness; DBP, diastolic blood pressure; FBG, fasting blood glucose; HbA1c, glycated hemoglobin; HDL-C, high-density lipoprotein cholesterol; LDL-C, low-density lipoprotein cholesterol; PH, postprandial hypertriglyceridemia; PN, postprandial normal triglyceride; RC, remnant cholesterol; SBP, systolic blood pressure; TC, total cholesterol; TG, triglycerides.

Chi-square tests were used to compare categorical variables; in contrast, *t*-tests analyzed normally distributed variables, and Mann–Whitney *U* tests were applied to non-normally distributed variables between the PN and PH groups.

The proportion of males was significantly higher in the PH group than in the PN group (*P* = 0.009). At baseline and follow-up, BMI, waist-to-hip ratio, fasting TG, RC, non-HDL-C levels, and cIMT were significantly higher in the PH group than in the PN group (*P* < 0.05). Additionally, HDL-C levels were significantly lower in the PH group (*P* < 0.05). At follow-up, HbA1c, TC, and LDL-C levels were also significantly higher in the PH group (*P* < 0.05). No significant differences were observed in age, SBP, or DBP between the two groups at baseline or follow-up (*P* > 0.05).

**Figure 1:**
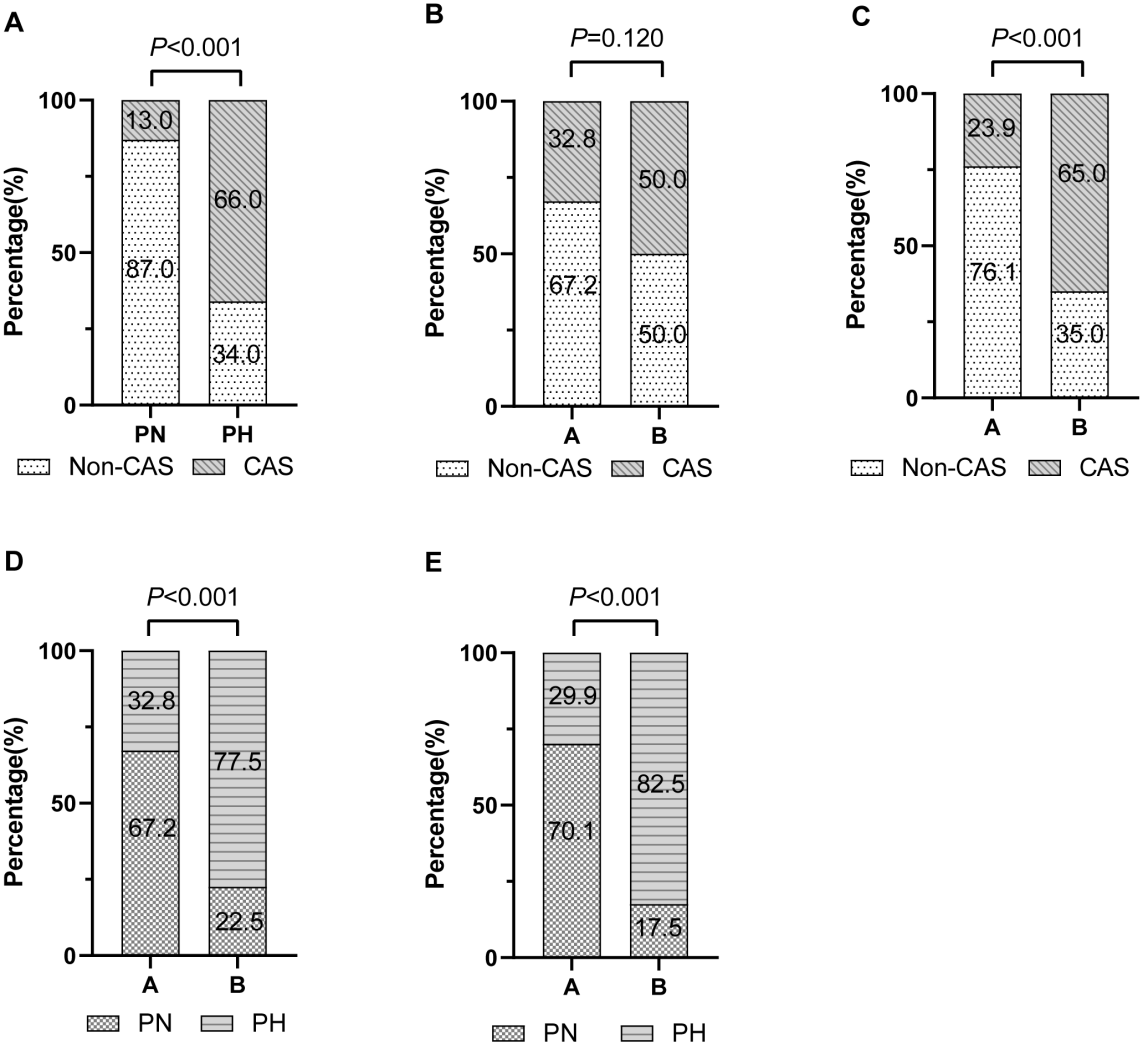
Percentage bar chart. **(A)** Proportion of carotid atherosclerosis (CAS) and Non-CAS participants in the postprandial normal triglyceride (PN) and postprandial hypertriglyceridemia (PH) groups. **(B, C)** Proportion of CAS participants in the A (fasting triglyceride < 1.2 mmol/L) and B (fasting triglyceride 1.2–1.7 mmol/L) groups at baseline and follow-up, respectively. **(D, E)** Proportion of PN and PH participants in the A and B groups at baseline and follow-up, respectively.

Among the participants, 42 (39.3%) developed CAS. As shown in **Figure 1A**, the proportion of participants with CAS in the PH group was 66.0%, significantly higher than the 13.0% observed in the PN group (*P* < 0.001).

### 3.2 Changes in blood lipids during OFTT in participants with different postprandial TG levels

**Figure 2:**
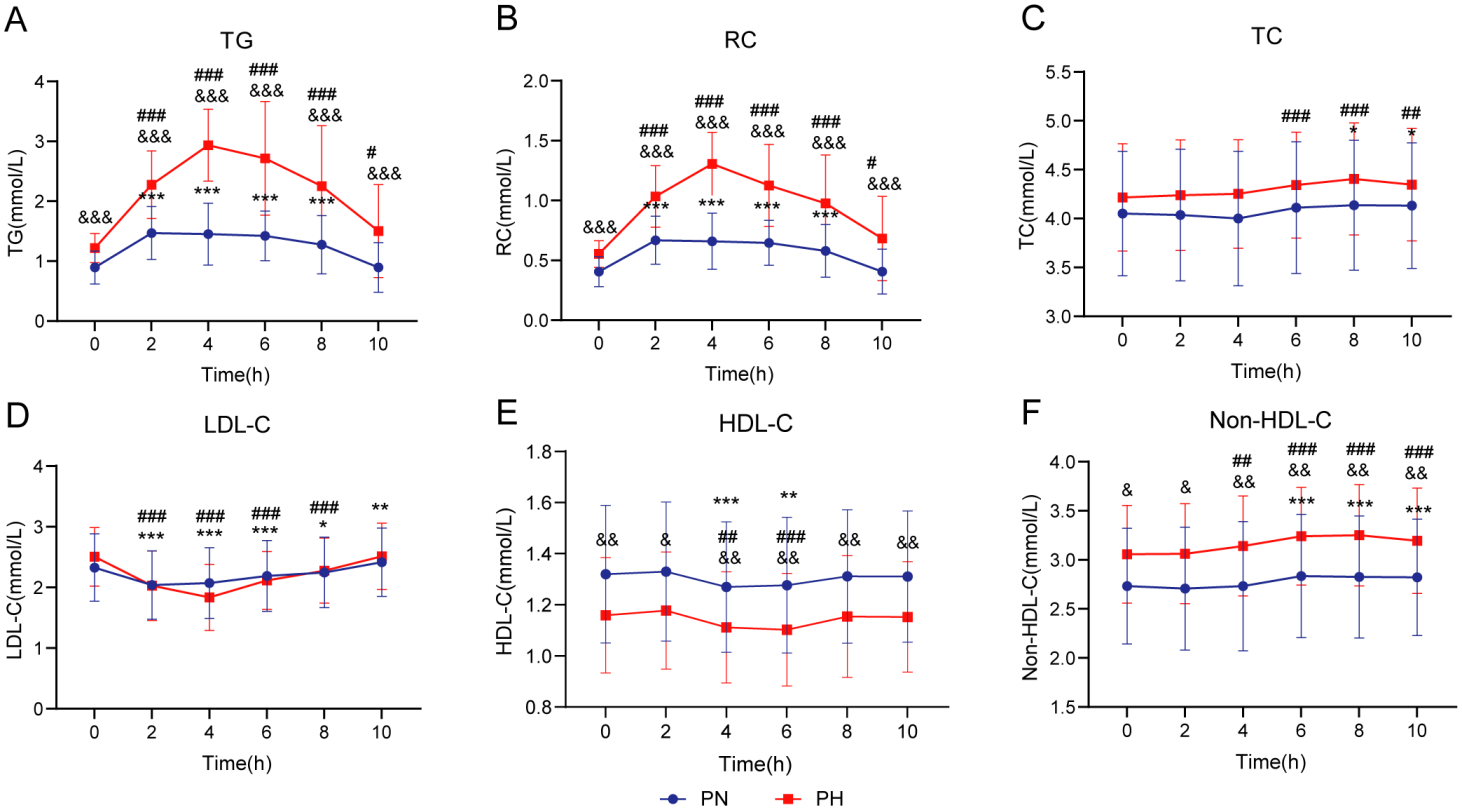
Lipid profile changes during oral fat tolerance tests among individuals stratified by postprandial triglyceride levels. **(A)** Triglyceride (TG). **(B)** Remnant cholesterol (RC). **(C)** Total cholesterol (TC). **(D)** Low-density lipoprotein cholesterol (LDL-C). **(E)** High-density lipoprotein cholesterol (HDL-C). **(F)** Non-HDL-C. Data are presented as mean ± standard deviation. Statistical significance is indicated as follows: ^*^ *P* < 0.05, ^**^ *P* < 0.01, and ^***^ *P* < 0.001 compared to the fasting level in the postprandial normal triglyceride (PN) group, ^#^ *P* < 0.05, ^##^ *P* < 0.01, and ^###^ *P* < 0.001 compared to the fasting level in the postprandial hypertriglyceridemia (PH) group, and ^&^ *P* < 0.05, ^&&^ *P* < 0.01, and ^&&&^ *P* < 0.001 compared to the corresponding time point in the PN group. Two-way repeated-measures analysis of variance was used to compare the effects of time and group on blood lipid levels during the oral fat tolerance tests, using the Šidák method for multiple comparisons.

**Figure 2** illustrates blood lipid changes during the OFTT in the PN and PH groups. At all time points, TG, RC, and non-HDL-C levels were significantly higher in the PH group than in the PN group (*P* < 0.05); in contrast, HDL-C levels were significantly lower (*P* < 0.05). No significant differences were observed in the TC and LDL-C levels between the groups at any time point (*P* > 0.05). Following a high-fat meal, both groups showed increased TG and RC levels. The PN group reached peak levels at 2 h, returning to fasting levels by 10 h. In contrast, the PH group peaked at 4 h, with levels remaining significantly elevated at 10 h (*P* < 0.05). In the PN group, postprandial TC levels were significantly higher than fasting levels at 8–10 h (*P* < 0.05); in contrast, the PH group exhibited this increase at 6–10 h (*P* < 0.05). The postprandial LDL-C levels reached their lowest at 2 h in the PN group and at 4 h in the PH group, with both groups returning to fasting levels by 10 h. For postprandial HDL-C levels, the lowest levels occurred at 4 h in the PN group and 6 h in the PH group, with both groups returning to fasting levels by 8 h. Postprandial non-HDL-C levels increased between 6 and 10 h in the PN group and between 4 and 10 h in the PH group, with neither group returning to fasting levels by 10 h.

### 3.3 Clinical characteristics of participants classified by CAS status

**Table 2** summarizes the clinical data of participants with and without CAS at baseline and follow-up. At both time points, age, BMI, fasting TG, RC levels, and cIMT were significantly higher in the CAS group than in the non-CAS group (*P* < 0.05). The baseline HDL-C level was significantly lower in the CAS group than in the non-CAS group (*P* = 0.007); however, no significant difference was observed at follow-up (*P* = 0.140). At follow-up, fasting blood glucose (FBG) and HbA1c levels were significantly higher in the CAS group; in contrast, no significant differences were found at baseline (*P* < 0.05). No statistically significant differences in sex, SBP, DBP, TC, LDL-C, or non-HDL-C levels were observed between the groups at either baseline or follow-up (*P* > 0.05).

**Table 2:** Clinical characteristics of the participants with and without CAS.

Variables	Baseline			Follow-up		
	Non-CAS (n = 65)	CAS (n = 42)	*P* value	Non-CAS (n = 65)	CAS (n = 42)	*P* value
Sex (Men, %)	29 (44.6)	23 (54.8)	0.408	29 (44.6)	23 (54.8)	0.408
Age (year)	46 (34, 50)	50 (44, 58)	0.002	52 (40, 56)	56 (50, 64)	0.002
SBP (mmHg)	120.71 ± 14.62	122.83 ± 13.82	0.455	125.28 ± 15.52	127.98 ± 14.22	0.366
DBP (mmHg)	75.00 (70.00, 80.00)	77.50 (70.25, 83.50)	0.112	77.00 (72.00, 84.00)	78.00 (72.50, 83.50)	0.442
BMI (kg/m^2^)	24.90 (22.10, 26.70)	26.15 (24.65, 28.45)	0.012	25.27 (22.74, 27.41)	26.39 (25.27, 28.47)	0.011
Waist-to-hip ratio	0.86 ± 0.08	0.87 ± 0.07	0.466	0.86 ± 0.07	0.88 ± 0.07	0.089
FBG (mmol/L)	5.29 ± 0.48	5.41 ± 0.52	0.232	5.16 (4.91, 5.65)	5.51 (5.25, 6.17)	0.005
HbA1c (%)	5.40 (5.35, 5.70)	5.60 (5.50, 5.70)	0.073	5.50 (5.30, 5.70)	5.60 (5.50, 5.90)	0.016
TG (mmol/L)	1.00 ± 0.32	1.14 ± 0.27	0.015	0.90 (0.69, 1.11)	1.29 (1.01, 1.74)	< 0.001
RC (mmol/L)	0.45 ± 0.14	0.52 ± 0.12	0.015	0.41 (0.31, 0.50)	0.59 (0.46, 0.79)	< 0.001
TC (mmol/L)	4.11 (3.50, 4.61)	4.22 (3.95, 4.56)	0.583	4.51 ± 0.85	4.66 ± 0.74	0.351
LDL-C (mmol/L)	2.38 (1.96, 2.83)	2.47 (2.24, 2.85)	0.234	2.69 ± 0.66	2.75 ± 0.65	0.651
HDL-C (mmol/L)	1.22 (1.11, 1.45)	1.12 (1.02, 1.22)	0.007	1.30 (1.17, 1.62)	1.25 (1.04, 1.46)	0.140
Non-HDL-C (mmol/L)	2.90 (2.36, 3.33)	3.05 (2.79, 3.39)	0.093	3.13 ± 0.73	3.36 ± 0.63	0.098
cIMT (mm)	0.55 (0.45, 0.60)	0.64 (0.46, 0.80)	0.005	0.60 (0.50, 0.80)	1.05 (0.80, 1.10)	< 0.001

BMI, body mass index; CAS, carotid atherosclerosis; cIMT, carotid intima-media thickness; DBP, diastolic blood pressure; FBG, fasting blood glucose; HbA1c, glycated hemoglobin; HDL-C, high-density lipoprotein cholesterol; LDL-C, low-density lipoprotein cholesterol; RC, remnant cholesterol; SBP, systolic blood pressure; TC, total cholesterol; TG, triglycerides.

Chi-square tests were used to compare categorical variables, *t*-tests analyzed normally distributed variables, and Mann–Whitney *U* tests were applied to non-normally distributed variables between the Non-CAS and CAS groups.

### 3.4 Changes in blood lipids during OFTT in participants classified by CAS status

**Figure 3:**
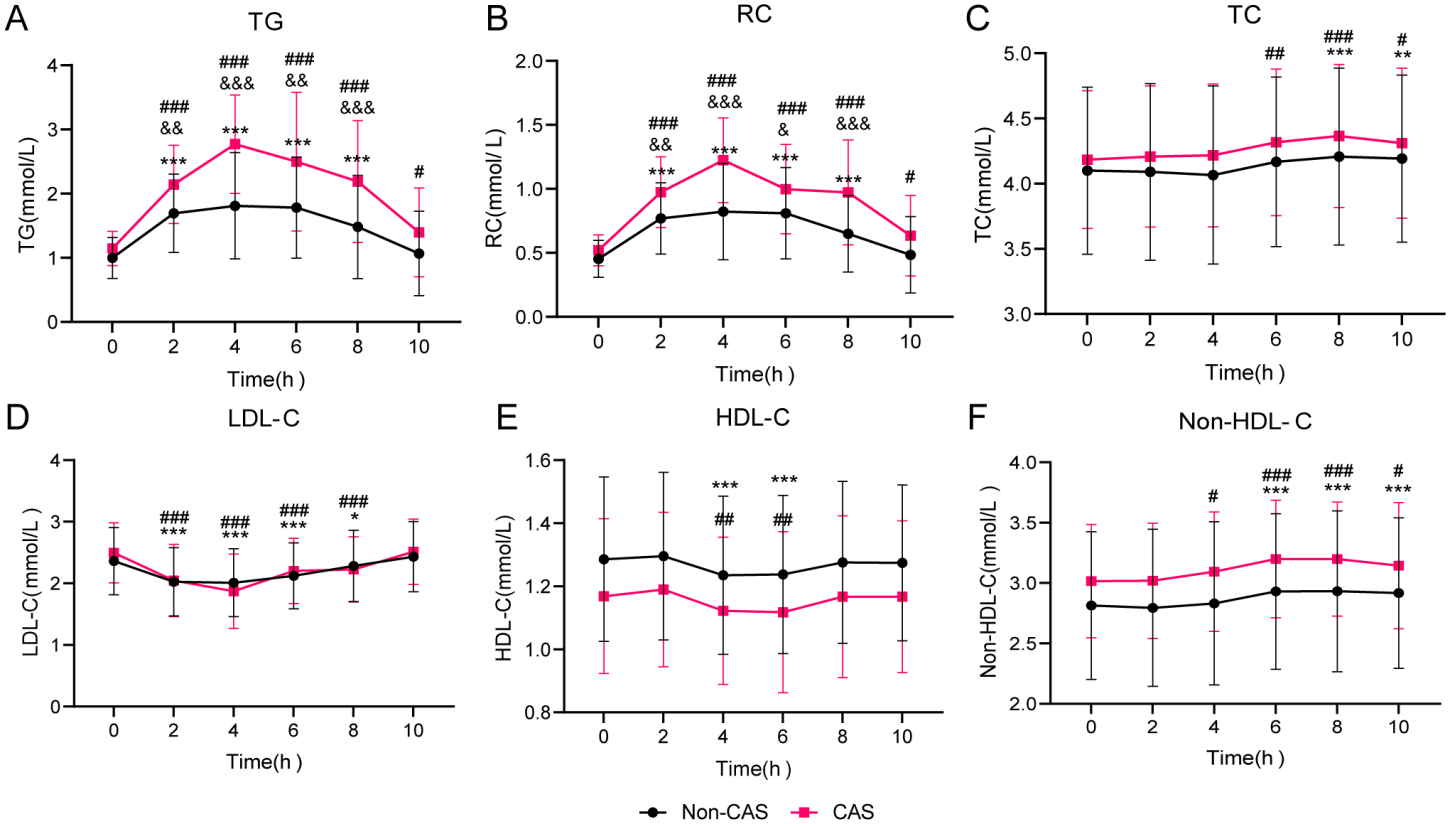
Lipid profile changes during oral fat tolerance tests among individuals stratified by carotid atherosclerosis (CAS) status. **(A)** Triglyceride (TG). **(B)** Remnant cholesterol (RC). **(C)** Total cholesterol (TC). **(D)** Low-density lipoprotein cholesterol (LDL-C). **(E)** High-density lipoprotein cholesterol (HDL-C). **(F)** Non-HDL-C. Statistical significance is indicated as follows: ^*^ *P* < 0.05, ^**^ *P* < 0.01, and ^***^ *P* < 0.001 compared to the fasting level in the Non-CAS group, ^#^ *P* < 0.05, ^##^ *P* < 0.01, and ^###^ *P* < 0.001 compared to the fasting level in the CAS group, and ^&^ *P* < 0.05, ^&&^ *P* < 0.01, and ^&&&^ *P* < 0.001 compared to the corresponding time point in the Non-CAS group. Two-way repeated-measures analysis of variance was used to compare the effects of time and group on blood lipid levels during the oral fat tolerance tests, using the Šidák method for multiple comparisons.

**Figure 3** illustrates the blood lipid changes during the OFTT in the non-CAS and CAS groups. Fasting TG and RC levels did not significantly differ between the groups. However, from 2 to 10 h postprandially, TG and RC levels were significantly higher in the CAS group than in the non-CAS group (*P* < 0.05). No significant differences in TC, LDL-C, HDL-C, or non-HDL-C levels were observed between the groups at any time point. Following the high-fat meal, both groups experienced increases in TG and RC levels, peaking at 4 h. In the non-CAS group, these levels returned to fasting levels by 8 h, whereas in the CAS group, they remained significantly elevated even at 10 h (*P* < 0.05). Postprandial TC levels were significantly higher than fasting levels from 8 to 10 h in the non-CAS group (*P* < 0.05) and from 6 to 10 h in the CAS group (*P* < 0.05). Postprandial LDL-C and HDL-C levels reached their lowest points at 4 h in both groups and returned to fasting levels within 10 h. Postprandial non-HDL-C levels increased from 6 to 10 h in the non-CAS group and from 4 to 10 h in the CAS group, with neither group returning to fasting levels by 10 h.

As detailed in **Table 3**, postprandial TG_max_ and RC_max_ values were significantly higher in the CAS group than in the non-CAS group (*P* < 0.001). Additionally, the proportion of individuals with TG_max_ > 2.5 mmol/L was significantly greater in the CAS group (*P* < 0.001).

**Table 3:** Comparison of TG_max_ and RC_max_ at any time after meals grouped by CAS.

Variables	Non-CAS	CAS	*P* value
TG_max_ (mmol/L)	2.05 (1.57, 2.68)	3.13 (2.69, 3.63)	< 0.001
RC_max_ (mmol/L)	0.93 (0.71, 1.22)	1.42 (1.22, 1.55)	< 0.001
TG_max_ > 2.5mmol/L	18 (27.7)	35 (83.3)	< 0.001

CAS, carotid atherosclerosis; RC_max_, maximum postprandial remnant cholesterol; TG_max_, maximum postprandial triglycerides.

Chi-square tests compared categorical variables, and Mann–Whitney *U* tests were applied to non-normally distributed variables between the Non-CAS and CAS groups.

### 3.5 Proportions of participants achieving optimal fasting TG levels in different groups

At baseline, there was no significant difference in the proportion of individuals with CAS across fasting TG groups (*P* = 0.120) (**Figure 1B**). However, at follow-up, the proportions of individuals with fasting TG levels ≥ 1.2 mmol/L were significantly higher in the CAS group than in the non-CAS group, compared with the proportions of those with fasting TG < 1.2 mmol/L (*P* < 0.001) (**Figure 1C**). Similarly, at both baseline and follow-up, the proportion of individuals with fasting TG levels ≥ 1.2 mmol/L was significantly greater in the PH group than in the PN group (*P* < 0.001) (**Figures 1D, E**).

### 3.6 Apolipoproteins and inflammatory marker levels grouped by CAS status

This study found that the CAS group had significantly higher levels of Apo B, Apo C3, hs-CRP, and IL-6 at both fasting and 4 h postprandially compared with the non-CAS group (*P* < 0.05). Additionally, within each group, 4-h postprandial levels of Apo C3 and IL-6 were significantly higher than the fasting levels (*P* < 0.05). Details are shown in **Table 4**.

**Table 4:** Comparison of apolipoprotein and inflammatory marker levels at fasting and 4-h postprandial, grouped by CAS.

Variables	0 h	4 h	*P* value
ApoB (mg/dL)			
Non-CAS	64.62 ± 14.78	62.32 ± 14.89	0.380
CAS	71.38 ± 12.75	69.31 ± 13.75	0.476
*P* value	0.016	0.016	
ApoC3 (mg/dL)			
Non-CAS	9.63 (7.12,12.63)	19.26 (16.97, 21.46)	< 0.001
CAS	12.05 (8.99,14.74)	24.67 (19.52, 27.47)	< 0.001
*P* value	0.006	< 0.001	
hs-CRP (mg/L)			
Non-CAS	1.85 (1.44, 2.06)	1.81 (1.53, 2.05)	0.972
CAS	2.94 (2.01, 3.23)	2.92 (1.93, 3.36)	0.929
*P* value	< 0.001	< 0.001	
IL-6 (pg/mL)			
Non-CAS	1.75 (1.45, 1.99)	2.56 (2.06, 2.95)	< 0.001
CAS	2.00 (1.57, 2.35)	3.01 (2.86, 3.52)	< 0.001
*P* value	0.016	< 0.001	

ApoB, apolipoprotein B; ApoC3, apolipoprotein C3; CAS, carotid atherosclerosis; hs-CRP, high-sensitivity C-reactive protein; IL-6, interleukin-6.

*t*-tests were used for normally distributed variables and Mann–Whitney *U* tests for non-normally distributed variables. These analyses compared the Non-CAS and CAS groups as well as the between 0-h and 4-h postprandial periods.

### 3.7 Relationship between postprandial lipid levels and CAS

Univariate logistic regression models were performed with CAS presence as the dependent variable and various baseline measurements as independent variables. Age had a significant effect on CAS (OR [95% CI] = 1.07 [1.03, 1.12], *P* = 0.001). Baseline BMI and HDL-C levels also significantly influenced CAS (OR [95% CI] = 1.12 [1.00, 1.24], *P* = 0.046; OR [95% CI] = 0.14 [0.03, 0.80], *P* = 0.027, respectively). Significant effects were also observed for baseline ApoB and ApoC3 levels (OR [95% CI] = 1.04 [1.01, 1.07], *P* = 0.019; OR [95% CI] = 1.17 [1.05, 1.31], *P* = 0.004, respectively) and for baseline hs-CRP and IL-6 levels (OR [95% CI] = 4.76 [2.45, 9.24], *P* < 0.001; OR [95% CI] = 2.85 [1.15, 7.03], *P* = 0.023, respectively). No statistically for waist-to-hip ratio, SBP, DBP, FBG, HbA1c, TC, LDL-C, and non-HDL-C levels (*P* > 0.05). Details are shown in **Table 5**.

**Table 5:** Univariate logistic regression of clinical variables with CAS at baseline.

Variables	OR (95% CI)	*P* value
Sex	1.50 (0.68, 3.31)	0.315
Age (year)	1.07 (1.03, 1.12)	0.001
BMI (kg/m^2^)	1.12 (1.00, 1.24)	0.046
Waist-to-hip ratio	1.22 (0.72, 2.08)	0.462
SBP (mmHg)	1.01 (0.98, 1.04)	0.451
DBP (mmHg)	1.04 (1.00, 1.08)	0.067
FBG (mmol/L)	1.64 (0.73, 3.70)	0.231
HbA1c (%)	1.75 (0.43, 7.10)	0.431
TC (mmol/L)	1.27 (0.66, 2.45)	0.478
LDL-C (mmol/L)	1.65 (0.77, 3.54)	0.197
HDL-C (mmol/L)	0.14 (0.03, 0.80)	0.027
Non-HDL-C (mmol/L)	1.93 (0.94, 3.96)	0.075
ApoB (mg/dL)	1.04 (1.01, 1.07)	0.019
ApoC3 (mg/dL)	1.17 (1.05, 1.31)	0.004
hs-CRP (mg/L)	4.76 (2.45, 9.24)	< 0.001
IL-6 (pg/mL)	2.85 (1.15, 7.03)	0.023

ApoB, apolipoprotein B; ApoC3, apolipoprotein C3; BMI, body mass index; CAS, carotid atherosclerosis; CI, confidence interval; DBP, diastolic blood pressure; FBG, fasting blood glucose; HbA1c, glycated hemoglobin; HDL-C, high-density lipoprotein cholesterol; hs-CRP, high-sensitivity C-reactive protein; IL-6, interleukin-6; LDL-C, low-density lipoprotein cholesterol; OR, odds ratio; SBP, systolic blood pressure; TC, total cholesterol.

The reference category for sex was female. All biochemical indicators were measured at fasting levels. A 0.1 unit increase was observed for the waist-to-hip ratio, LDL-C, and HDL-C values.

The logistic regression analysis of TG and RC levels in relation to CAS is presented in **Table 6**. In Model 1 (crude model), all variables (fasting TG, TG_4h_, TG_max_, fasting RC, RC_4h_, and RC_max_) were significantly associated with CAS (*P* < 0.05). Model 2, adjusted for baseline age and BMI, also showed significant associations for all variables (*P* < 0.05). In Model 3, further adjusted for fasting HDL-C, TG_4h_, TG_max_, RC_4h_, and RC_max_ retained significant associations with CAS (*P* < 0.001); in contrast, fasting TG and RC did not (*P* = 0.061). In Model 4, which additionally adjusted for ApoB, ApoC3, hs-CRP, and IL-6 levels, TG_4h_, TG_max_, RC_4h_, and RC_max_ remained significantly associated with CAS (*P* < 0.01); in contrast, fasting TG and RC did not (*P* = 0.200).

**Table 6:** Logistic regression of TG and RC levels with CAS.

Variables	Model 1		Model 2		Model 3		Model 4	
	OR(95%CI)	*P* value	OR(95%CI)	*P* value	OR(95%CI)	*P* value	OR(95%CI)	*P* value
Fasting TG	1.18 (1.03, 1.35)	0.017	1.19 (1.03, 1.40)	0.022	1.16 (1.00, 1.37)	0.061	1.15 (0.95, 1.40)	0.200
TG_4h_	1.15 (1.08, 1.22)	< 0.001	1.16 (1.09, 1.24)	< 0.001	1.15 (1.08, 1.23)	< 0.001	1.17 (1.08, 1.28)	< 0.001
TG_max_	1.13 (1.07, 1.19)	< 0.001	1.14 (1.07, 1.22)	< 0.001	1.12 (1.06, 1.20)	< 0.001	1.11 (1.04, 1.21)	0.006
Fasting RC	1.44 (1.07, 1.94)	0.017	1.48 (1.07, 2.10)	0.022	1.39 (0.99, 2.00)	0.061	1.35 (0.90, 2.10)	0.200
RC_4h_	1.34 (1.18, 1.51)	< 0.001	1.36 (1.20, 1.59)	< 0.001	1.34 (1.17, 1.56)	< 0.001	1.39 (1.16, 1.71)	< 0.001
RC_max_	1.35 (1.18, 1.54)	< 0.001	1.39 (1.21, 1.65)	< 0.001	1.37 (1.18, 1.61)	< 0.001	1.32 (1.11, 1.62)	0.003

ApoB, apolipoprotein B; ApoC3, apolipoprotein C3; BMI, body mass index; CAS, carotid atherosclerosis; HDL-C, high-density lipoprotein cholesterol; hs-CRP, high-sensitivity C-reactive protein; IL-6, interleukin-6; RC, remnant cholesterol; RC_max_, maximum postprandial RC; RC_4h_, 4h-postprandial RC; TG, triglycerides; TG_max_, maximum postprandial TG; TG_4h_, 4h-postprandial TG.

Model 1: Crude model. Model 2: Adjusted for age and BMI at baseline. Model 3: Adjusted for age, BMI, and fasting HDL-C levels at baseline. Model 4: Adjusted for age, BMI, fasting HDL-C, ApoB, ApoC3, hs-CRP, and IL-6 levels at baseline. For each unit increase, TG, RC, and HDL-C values increased by 0.1.

### 3.8 Subgroup analysis

Subgroup analyses (**Table 7**) were conducted by sex, age (< 47 and ≥ 47 years), and BMI (< 25 and ≥ 25 kg/m^2^), using fasting TG, TG_4h_, TG_max_, fasting RC, RC_4h_, and RC_max_ as predictors in the regression equations. The results indicated that sex, age, and BMI did not significantly modify the relationships between these lipid parameters and CAS (*P* for interaction > 0.05).

**Table 7:** Subgroup analysis of TG and RC levels with CAS.

TG				RC			
Subgroups	OR(95%CI)	*P* value	*P* for interaction	Subgroups	OR(95%CI)	*P* value	*P* for interaction
Fasting TG				Fasting RC			
Sex			0.462	Sex			0.462
Women	1.25 (0.99, 1.58)	0.059		Women	1.64 (0.98, 2.75)	0.059	
Men	1.12 (0.95, 1.34)	0.182		Men	1.30 (0.89, 1.89)	0.182	
Age(year)			0.528	Age(year)			0.528
≥47	1.23 (1.02, 1.48)	0.030		≥47	1.58 (1.05, 2.38)	0.030	
<47	1.12 (0.91, 1.38)	0.266		<47	1.30 (0.82, 2.04)	0.266	
BMI(kg/m^2^)			0.336	BMI(kg/m^2^)			0.336
≥25	1.23 (1.03, 1.47)	0.023		≥25	1.58 (1.07, 2.33)	0.023	
<25	1.07 (0.84, 1.34)	0.594		<25	1.15 (0.69, 1.92)	0.594	
TG_4h_				RC_4h_			
Sex			0.327	Sex			0.516
Women	1.12 (1.03, 1.21)	0.006		Women	1.28 (1.07, 1.53)	0.006	
Men	1.19 (1.08, 1.31)	< 0.001		Men	1.40 (1.16, 1.68)	< 0.001	
Age(year)			0.737	Age(year)			0.884
≥47	1.17 (1.07, 1.27)	< 0.001		≥47	1.37 (1.15, 1.63)	< 0.001	
<47	1.14 (1.05, 1.25)	0.003		<47	1.34 (1.10, 1.63)	0.003	
BMI(kg/m^2^)			0.589	BMI(kg/m^2^)			0.888
≥25	1.13 (1.05, 1.21)	< 0.001		≥25	1.31 (1.12, 1.52)	< 0.001	
<25	1.17 (1.05, 1.30)	0.005		<25	1.33 (1.06, 1.67)	0.013	
TG_max_				RC_max_			
Sex			0.942	Sex			0.503
Women	1.13 (1.04, 1.22)	0.004		Women	1.30 (1.09, 1.55)	0.004	
Men	1.13 (1.05, 1.22)	0.002		Men	1.43 (1.15, 1.78)	0.001	
Age(year)			0.200	Age(year)			0.391
≥47	1.10 (1.03, 1.17)	0.003		≥47	1.29 (1.10, 1.52)	0.002	
<47	1.19 (1.07, 1.32)	0.002		<47	1.47 (1.15, 1.87)	0.002	
BMI(kg/m^2^)			0.704	BMI(kg/m^2^)			0.890
≥25	1.11 (1.04, 1.18)	0.003		≥25	1.32 (1.12, 1.55)	0.001	
<25	1.13 (1.03, 1.25)	0.011		<25	1.34 (1.06, 1.70)	0.014	

BMI, body mass index; CAS, Carotid atherosclerosis; CI, confidence interval; OR, odds ratio; RC, remnant cholesterol; RC_max_, maximum postprandial RC; RC_4h_, 4h-postprandial RC; TC, total cholesterol; TG, triglycerides; TG_max_, maximum postprandial TG; TG_4h_, 4h-postprandial TG.

TG and RC increased by 0.1 per unit.

## 4 Discussion

This follow-up study included 107 participants with normal fasting lipid levels and no CAS at baseline. Participants were classified into PN and PH groups based on their postprandial TG levels during the OFTT. Compared to the PN group, the PH group exhibited significantly higher levels of TRL, including TG and RC, in both fasting and postprandial states. Over 6 years, the PH group demonstrated a significantly higher incidence of CAS. Although fasting lipid levels did not differ between the CAS and non-CAS groups, the CAS group showed considerably elevated postprandial TRL levels. A similar study in the Chinese population compared 60 patients with coronary atherosclerotic heart disease and 30 healthy controls. It found that TG and RC levels peaked 4 h after consuming a 50 g fat meal, with patients showing significantly higher fasting and postprandial TG and RC levels than controls (23). Unlike this study, which examined advanced disease, our research focused on early atherosclerosis. The findings revealed that postprandial lipid differences, detectable via the OFTT, may serve as early predictive markers, even when fasting lipid levels remain normal. A Japanese follow-up study involving 115 patients with type 2 diabetes similarly identified postprandial TG levels as an independent risk factor for CAS over 1 year (24). However, that study included participants with baseline lipid abnormalities, hypertension, and medication use; in contrast, our study rigorously excluded such confounding factors, enhancing the reliability of the results.

The 2021 European Atherosclerosis Society consensus identifies optimal fasting TG level as < 1.2 mmol/L and borderline levels as 1.2–1.7 mmol/L (6). Stratification by postprandial TG levels (>2.5 mmol/L) during the baseline OFTT showed a significant increase in the PH group among those with borderline fasting TG levels. This aligns with the consensus that TRL and remnants tend to accumulate in plasma when fasting TG levels exceed 1.2 mmol/L. Notably, there were no significant differences in CAS prevalence across fasting TG groups at baseline; however, CAS incidence was significantly higher in the borderline TG group at follow-up. These findings suggest that fasting TG levels alone are insufficient to predict CAS risk and highlight the utility of postprandial TG assessment via the OFTT. The OFTT provides valuable insights into the development of CAS. Studies show that approximately 75% of individuals with fasting TG levels between 1.0 and 1.7 mmol/L have postprandial TG levels exceeding 2.5 mmol/L, highlighting the utility of the OFTT in this population (25).

The present study identified elevated postprandial TG and RC levels as independent risk factors for CAS, with TG_max_ and RC_max_ values significantly higher in the CAS group than in the non-CAS group. These results indicate impaired TRL clearance and remnant metabolism in the CAS population. Consistent with Zilversmit’s 1979 hypothesis that atherosclerosis forms postprandially (26, 27), this study provides robust longitudinal evidence supporting this theory. The baseline OFTT in this study revealed that the primary lipid changes in the postprandial state occurred in TG and RC. When TG levels were < 4.0 mmol/L, RC, primarily calculated from TG levels, exhibited similar postprandial patterns as TG. In contrast, traditional atherogenic indicators such as TC, HDL-C, and non-HDL-C showed no significant changes postprandially, compared to the fasting levels. LDL-C, calculated using the Friedewald formula, displayed an opposite trend—initially decreasing and then increasing—due to the rise and subsequent fall of postprandial TRL.

Extensive cross-sectional research has linked postprandial TRL to CAS and ASCVD. These studies, influenced by diet and the timing of postprandial blood sample collection, can be broadly classified into two categories: OFTT studies using a standard high-fat meal (where fat provides ≥ 50% of total calories) and non-fasting studies conducted under normal dietary conditions. This latter approach is analogous to the oral 75-g glucose tolerance test or random blood sugar measurements used in diabetes diagnosis. Non-fasting blood lipid testing is widely used in large-scale epidemiological studies. The Copenhagen City Heart Study and the Copenhagen General Population Study linked non-fasting TG and RC levels to elevated risks of ischemic heart disease, myocardial infarction, and overall mortality (28–30). Similarly, the U.S. Women’s Health Study identified non-fasting TG as an independent cardiovascular risk factor (7). For patients with coronary artery disease, RC is positively correlated with the risks of all-cause and cardiovascular mortality, as well as major adverse cardiovascular events (31). In Chinese patients with coronary heart disease, both fasting and non-fasting lipid profiles are associated with long-term major adverse cardiovascular events. In addition to fasting LDL-C, low non-fasting HDL-C may also independently predict cardiovascular events (32). The rs662799 locus of apolipoprotein A5 is significantly linked to ASCVD and regulates TG levels, suggesting a causal relationship between the TG metabolic pathway and ASCVD (33). In hypertriglyceridemia, cholesteryl ester transfer protein is activated, promoting the exchange of TG and cholesteryl esters between very-low-density lipoproteins and LDL-C, leading to increased TG levels in LDL-C (34). Elevated TG levels in LDL-C are associated with a higher risk of ASCVD and its components (22).

In small-scale OFTT studies, elevated postprandial TG has been linked to early cardiovascular changes. For example, Grønholdt et al. (35) found that postprandial TRLs were associated with carotid plaque appearance in patients with carotid artery disease. Other studies have shown that TG_4h_ values are a stronger predictor of early CAS than fasting TG or LDL-C levels (36–38). Postprandial lipemia is also associated with endothelial dysfunction, an early marker of atherosclerosis (39, 40). Delay in TG peaks, compared to normal glucose tolerance, are associated with impaired glucose tolerance and type 2 diabetes. Additionally, postprandial TG and ApoB concentrations are positively correlated with cIMT and inversely correlated with the ankle-brachial index (41). In a trial by Mena-Vazquez et al. (42), both rheumatoid arthritis and healthy control groups consumed a mixed meal containing 50 g of fat and 775 kcal. The study found that elevated levels of TG_4h_ were positively correlated with the presence of carotid atherosclerotic plaques and increased inflammatory mediators in the rheumatoid arthritis group.

Inflammatory markers, such as IL-6, impair lipoprotein lipase activity, reducing TRL clearance and increasing plasma TG levels (43). This study found elevated fasting and postprandial levels of IL-6 and hs-CRP in the CAS group, suggesting a chronic inflammatory state (44, 45). Additionally, elevated ApoB and ApoC3 levels were observed in the CAS group, with ApoC3 levels higher postprandially than at fasting. These findings align with previous research linking ApoB and ApoC3 to increased ASCVD risk (26, 41). In this study, TG_4h_, TG_max_, RC_4h_, and RC_max_ emerged as independent CAS predictors, even after adjusting for fasting lipid levels, ApoB, ApoC3, hs-CRP, and IL-6. These findings support the potential of postprandial TRL as early biomarkers of CAS.

This study has several strengths. First, strict inclusion criteria excluded confounding factors such as diabetes, hypertension, and baseline lipid abnormalities, ensuring robust results. Second, the standardized OFTT protocol enhanced the reliability of postprandial lipid measurements. Third, the longitudinal design allowed for the identification of metabolic disturbances preceding CAS onset.

However, the study also has some limitations. First, this study was conducted with a relatively small sample size and in a single center. This limitation may have affected the statistical power of subgroup analyses, potentially limiting the generalizability of the findings to other populations. Additionally, the absence of annual follow-ups prevented precise determination of CAS onset. Lifestyle factors during the follow-up period, such as diet and exercise, were not monitored, potentially introducing unmeasured confounders (46).

In conclusion, the OFTT demonstrated significant predictive value for CAS, with postprandial TRL, including TG and RC, serving as early markers in individuals with normal fasting lipid levels. These findings underscore the importance of postprandial lipid metabolism as a screening tool for early atherosclerotic changes. Further research is needed to validate these findings in larger, multicenter cohorts and to explore interventions targeting postprandial lipid disorders for ASCVD prevention.

## Data Availability

The original contributions presented in the study are included in the article/Supplementary Material. Further inquiries can be directed to the corresponding author.
